# A proteomic time course through the differentiation of human induced pluripotent stem cells into hepatocyte-like cells

**DOI:** 10.1038/s41598-019-39400-1

**Published:** 2019-03-01

**Authors:** Tracey Hurrell, Charis-Patricia Segeritz, Ludovic Vallier, Kathryn S. Lilley, Allan D. Cromarty

**Affiliations:** 1 Department of Pharmacology, Faculty of Health Sciences, School of Medicine, University of Pretoria, Private Bag X323, Arcadia, 0007 South Africa; 2 Wellcome–MRC Cambridge Stem Cell Institute, Anne McLaren Laboratory, University of Cambridge, Cambridge, CB2 0SZ UK; 3 University of Cambridge, Robinson Way, Cambridge, CB2 0SZ United Kingdom; 4 Wellcome Trust Sanger Institute, Hinxton, United Kingdom; 5 Cambridge Centre for Proteomics, Department of Biochemistry, University of Cambridge, Tennis Court Road, Cambridge, CB2 1QR United Kingdom

## Abstract

Numerous *in vitro* models endeavour to mimic the characteristics of primary human hepatocytes for applications in regenerative medicine and pharmaceutical science. Mature hepatocyte-like cells (HLCs) derived from human induced pluripotent stem cells (hiPSCs) are one such *in vitro* model. Due to insufficiencies in transcriptome to proteome correlation, characterising the proteome of HLCs is essential to provide a suitable framework for their continual optimization. Here we interrogated the proteome during stepwise differentiation of hiPSCs into HLCs over 40 days. Whole cell protein lysates were collected and analysed using stabled isotope labelled mass spectrometry based proteomics. Quantitative proteomics identified over 6,000 proteins in duplicate multiplexed labelling experiments across two different time course series. Inductive cues in differentiation promoted sequential acquisition of hepatocyte specific markers. Analysis of proteins classically assigned as hepatic markers demonstrated trends towards maximum relative abundance between differentiation day 30 and 32. Characterisation of abundant proteins in whole cells provided evidence of the time dependent transition towards proteins corresponding with the functional repertoire of the liver. This data highlights how far the proteome of undifferentiated precursors have progressed to acquire a hepatic phenotype and constructs a platform for optimisation and improved maturation of HLC differentiation.

## Introduction

The liver performs many metabolic processes including the detoxification of endogenous and xenobiotic compounds, which makes it a major contributor to high drug attrition rates^1^. Beyond the liver’s cellular diversity, intricate biophysical and biochemical cues, extracellular matrix, and local distribution of secreted factors contribute to the complexity of the liver^2^. Freshly isolated primary human hepatocytes (PHH) remain the “*gold standard*” for functional hepatocytes as liver-specific gene expression deteriorates with cryopreservation or plating^3,4^. However, improvements in the utilization of primary cells have negated some of these concerns^5–7^. Due to the historical limitations of PHHs, hepatocytes models have been developed to meet the challenges of logistics, practicality, complexity, reliability, reproducibility, and biological context of the liver *in vitro*^8^. Induced pluripotent stem cells (iPSCs) have revolutionized developmental biology and provide a source of self-renewing cells for *in vitro* models^9–11^.  Embryonic development is highly co-ordinated and provides a guideline for *in vitro* hepatocyte differentiation which can be mimicked using a multistage differentiation cascade through endoderm, anterior definitive endoderm, hepatocyte commitment, and hepatocyte maturation^12–14^. Differentiation to hepatocyte-like cells (HLC) begins with endoderm induction which typically makes use of a standard combination of growth factors. However, variation in the specification, extracellular matrices, culture format, and duration of culture influence differentiation kinetics^14–21^. Transcripts, protein expression, and functional activity of differentiated HLCs reportedly reflect a foetal phenotype^22,23^. Such limitations in differentiation are being addressed by 3D culture techniques in scalable technologies using spheroids^24^ and liver-on-a-chip^25^ approaches. However, in order for HLCs to be used as a surrogate for mature hepatocytes, robust phenotypic and functional characterisation is continually required. Despite specific mRNAs clusters suggested to define the hepatic phenotypic^13,17^, the changes in the proteome during differentiation are less rigorously investigated compared to the transcriptome. This study provides two proteomic time courses through HLC differentiation and illustrates the ability to trace hepatic marker proteins throughout *in vitro* differentiation. Isobaric tagging (TMT/iTRAQ) was employed, which allows for direct ratiometric comparison of independently collected samples and enables the relative quantification of large numbers of abundant proteins^26^. Using this quantitative proteomics approach, the lengths to which HLCs have progressed from undifferentiated precursors and the time dependent changes in relative abundance of proteins corresponding to the functional repertoire of hepatocytes was demonstrated.

## Results

### Differentiation of hepatocyte-like cells

**Figure 1:**
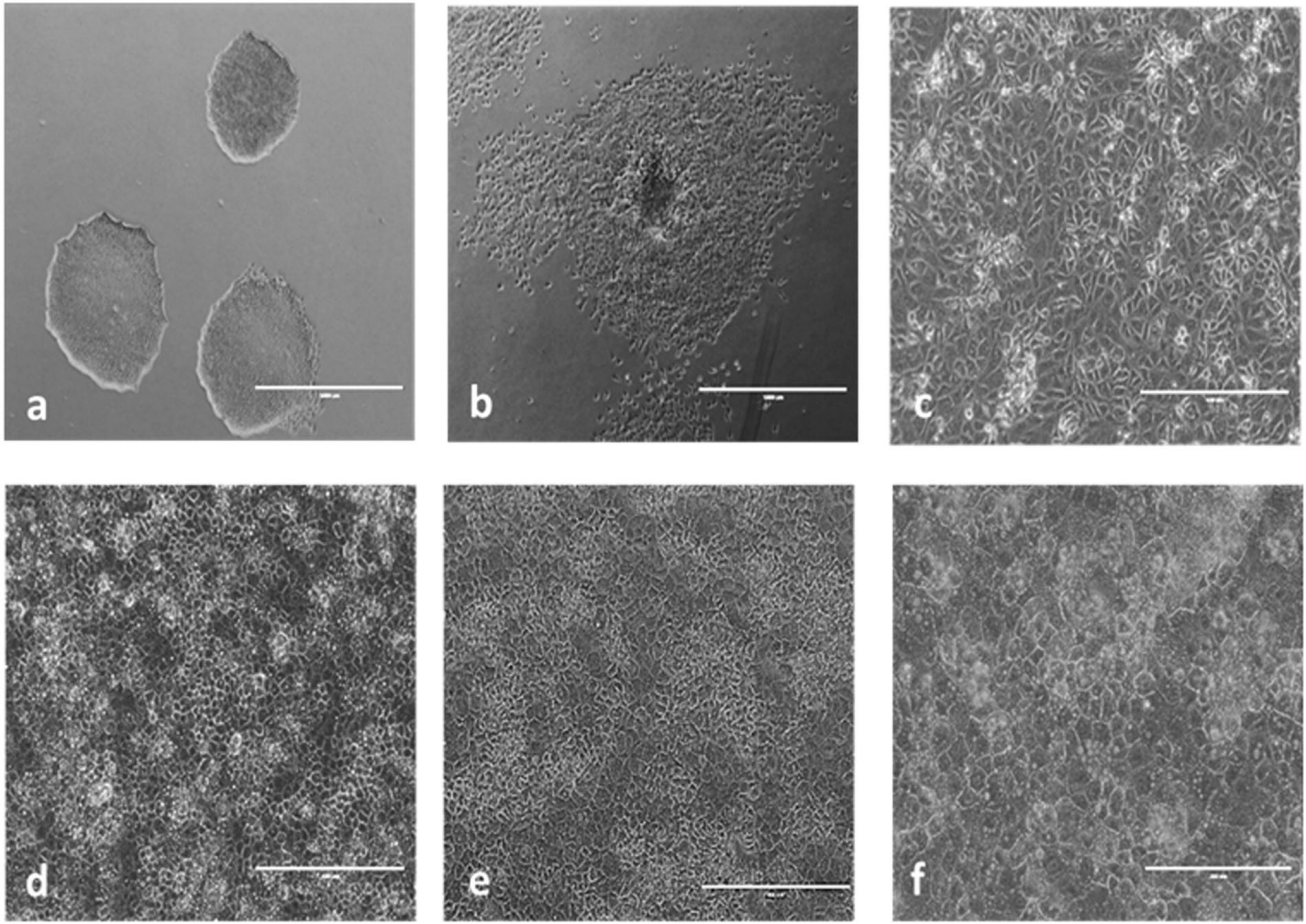
Phase contrast images of cell morphology through differentiation (EVOS FL Cell Imaging System, scale bar: 1000 µm). (**a**) Human iPSCs (not at differentiation density), (**b**) Anterior definitive endoderm at day 6, (**c**) Hepatic endoderm at day 9, (**d**) Hepatocyte-like cell maturation at day 16, (**e**) Hepatocyte-like cell maturation at day 25, and (**f**) HLCs at day 35.

Differentiation was observed morphologically (Fig. 1) as hiPSCs transition to anterior definitive endoderm which then acquire an epithelial-like morphology characteristic of hepatic progenitors, and finally mature to distinctly boundaried HLCs. RNA expression of hepatic markers albumin, A1AT, AFP, HNF4α as well as metabolizing enzymes CYP3A5 and CYP3A7 on day 35 of HLC differentiation was compared to hiPSCs and PHH donor samples (Supplementary 2: Fig. S2). Although expression did not rival that of PHHs, HLCs had increased mRNA expression compared to hiPSCs which was considered sufficiently representative of a transition to hepatic progenitors. Samples throughout six independent differentiations were harvested for various proteomic time courses.

### Overview of protein expression and principal component analysis

**Figure 2:**
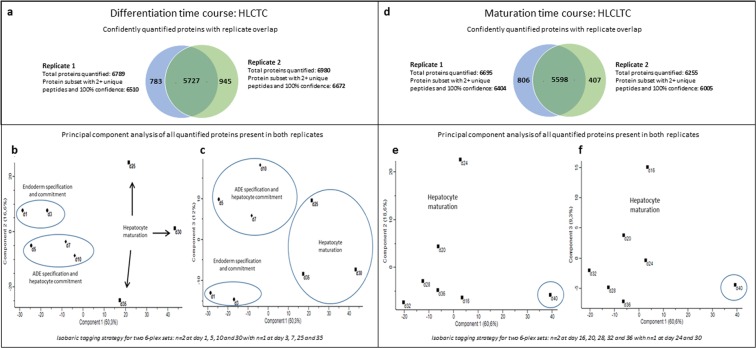
Overview of protein expression and PCA of the complete differentiation time course (HLCTC) and maturation time course (HLCLTC). (**a**) Number of proteins quantified per replicated in HLCTC with the corresponding overlap in commonly quantified proteins, (**b**) PCA comparing Component 1 and 2 during the HLCTC, (**c**) PCA comparing Component 1 and 3 during the HLCTC, (**d**) Number of proteins quantified per replicated in HLCLTC with the corresponding overlap in commonly quantified proteins, (**e**) PCA comparing Component 1 and 2 during the HLCLTC, and (**f**) PCA comparing Component 1 and 3 during the HLCLTC.

Relative quantification of proteins was determined across independent biological replicates with each replicate undergoing 30 hours of mass spectrometry to generate the protein cohorts described. Replicates 1 and 2 of the complete differentiation time course (HLCTC) from day 1 to day 35, identified 6,789 and 6,980 proteins respectively (Supplementary 1: Default report HLCTC replicate 1 and replicate 2). Filtering for protein group(s) with two or more unique peptides and a 100% confidence constrained the dataset to 5,727 proteins (Fig. 2a and Supplementary 1: HLCTC Replicate overlap). Within the maturation time course (HLCLTC) from day 16 to day 40, identified 6,695 and 6,255 proteins were in replicates 1 and 2 respectively (Supplementary 1: Default report HLCLTC replicate 1 and replicate 2) which constrained to 5,598 proteins when filtered (Fig. 2d and Supplementary 1: HLTLTC Replicate overlap). Principal component analysis (PCA) was done to reduce the redundancy of related data properties and summarize the data using the best linear combinations. These plots (Fig. 2b and c) achieved grouping of endoderm specification and commitment (day 1 and day 3) compared to anterior definitive endoderm specification and hepatocyte maturation (day 5, day 7, and day 10). Hepatocyte maturation (day 25, day 30, and day 35) was clearly segregated from the early time points in Component 1. PCA Component 1, which accounted for 50.3% of the variance, was able to distinguish HLCs from their undifferentiated precursors. Components 2 and 3 were able to distinguish the transition from iPSCs into endoderm. The lack of homogeneity in Components 2 and 3 at day 25, 30, and 35 could be due to the concomitant existence of other cell types during differentiation as HLCs were not specifically isolated from the total cellular cohort prior to analysis. However, under optimal conditions this differentiation protocol produces HLCs with more than 85% co-expression of albumin and A1AT from day 20^27^. In the maturation time course Component 1 made up 60.6% of the variance with the main distinction being to separate day 40 from the rest of the time points (Fig. 2e and f). While technical replicates of mass spectrometry can impact proteome coverage, this was not conducted as the protein identification was robust with the results of hierarchical clustering and PCA being able to demonstrate biological significance and not technical features of the labelled proteomics approach.

### Hierarchical clustering and hepatic markers throughout differentiation

**Figure 3:**
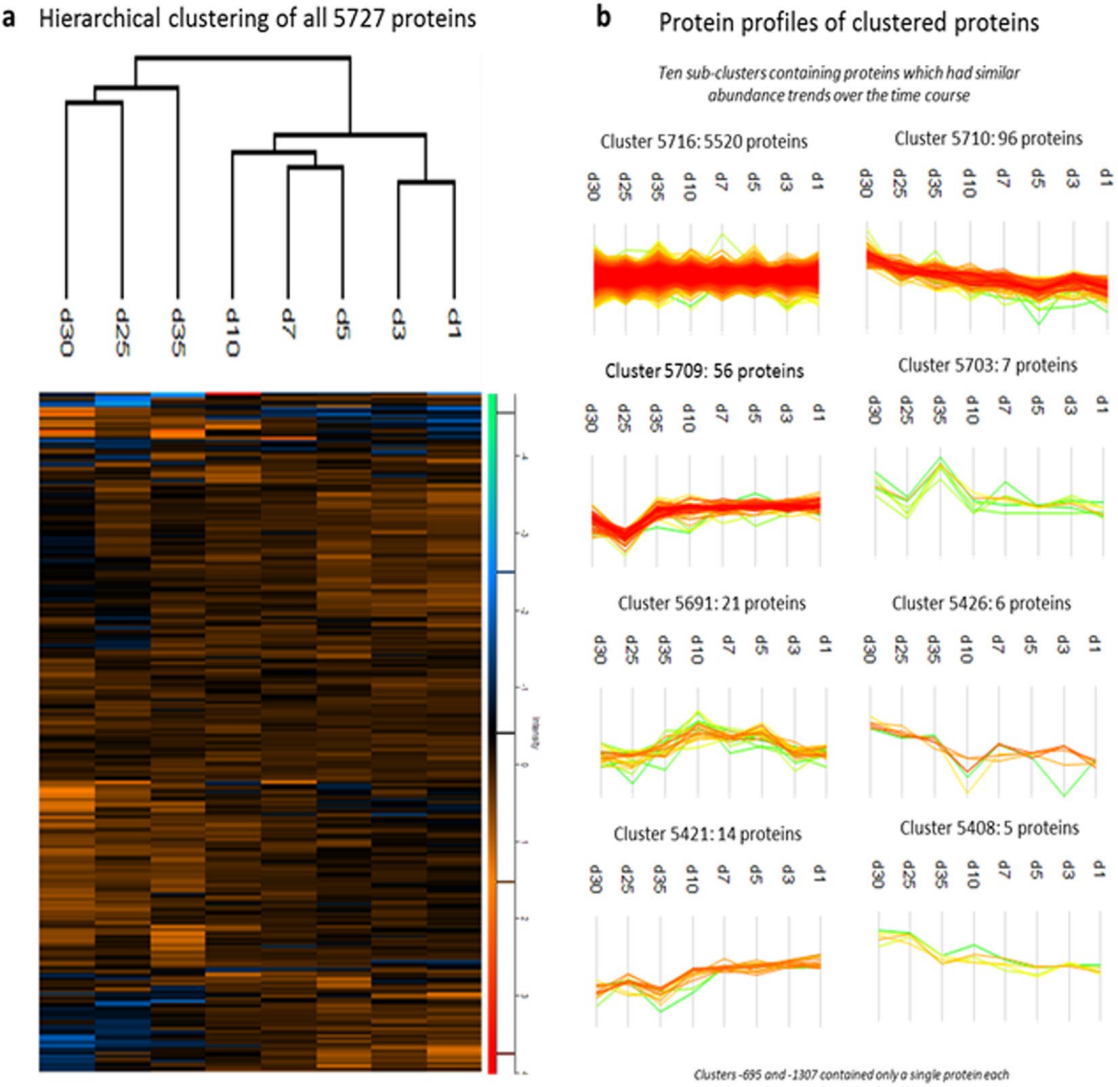
Hierarchical clustering and protein profiling throughout HLC differentiation. (**a**) Hierarchical clustering through HLC differentiation and (**b**) Clusters with profiles of proteins which have similar abundance changes over time.

Hierarchical clustering of samples across the complete differentiation time course generally co-segregated according to the differentiation phase with the major nodes distinguishing samples from day 1 to 10, and day 25 to 35 (Fig. 3a). Protein profiles highlighted similarities across a large proportion of the proteome but produced protein cohorts which increased or decreased over time (Fig. 3b). This provided proteins to scrutinize for insight into differentiation (Supplementary 1: HLCTC Hierarchical clustering and HLCTC generic clustering).

Schwartz *et al*. summarised the expected expression patterns throughout the differentiation cascade which distinguishes various cellular phenotypes (Supplementary 2: Table S2). Theses, in addition to proteins described by others provided the basis for hepatocyte characterisation^12–14,17,21,22,28,29^. Proteins governing transitions from pluripotency (NANOG, OCT4, SOX2 and SSEA3/4) were not detected, which could be expected as no nuclear enrichment was employed during protein extraction from whole cells. Despite lacking characteristic pluripotency markers, abundant proteins in hiPSCs included proteins associated with multicellular organismal development (lymphoid-specific helicase), G2/M transition (telomeric repeat-binding factor 1) and spindle-assembly checkpoint signalling. SOX17 modulates differentiation via WNT3A, which governs the development of regionalized endoderm, and was most abundant at day 5 suggestive of the transition into definitive endoderm. Additionally, neural cell adhesion molecule-1 (NCAM1), which is responsible for the organogenesis of mesodermal and endodermal derivatives, was the most abundant during foregut endoderm formation. Hepatic endoderm samples were rich in proteins involved in multicellular organismal development such as protein shisa-2 and vang-like protein 1 homolog.

**Figure 4:**
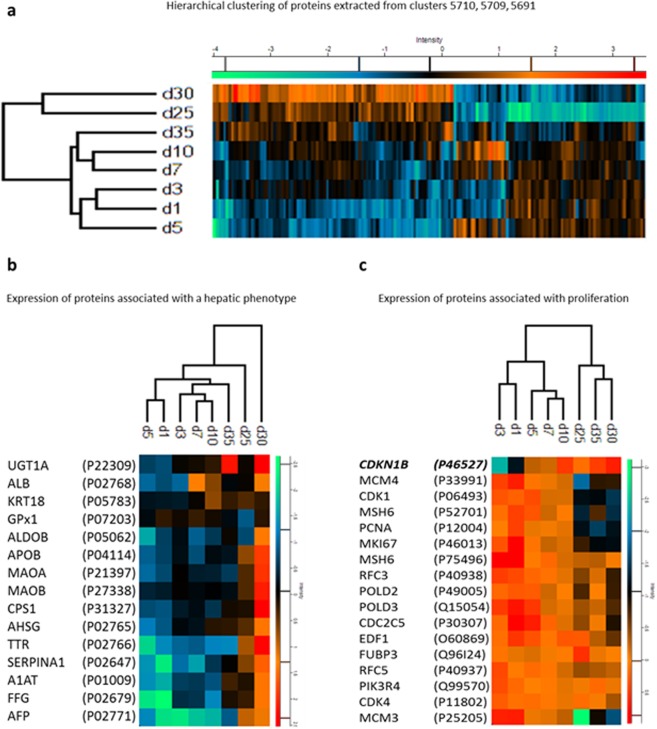
Hierarchical clustering of protein subsets from HLC differentiation. (**a**) Hierarchical clustering of Clusters 5710, 5691, and 5709 which accounted for the majority of variance throughout the time course, (**b**) Expression profiles of proteins marking a hepatic phenotype, and (**c**) Expression profiles of proteins marking proliferation alongside cyclin-dependent kinase inhibitor 1B (CDKI1B) which is involved in G1 arrest.

Subsets of the clustered proteins (Cluster 5710 with 96 proteins, Cluster 5691 with 21 proteins, and Cluster 5709 with 56 proteins) which accounted for the most dynamic variance were extracted and hierarchical clustering was re-performed. This clustered day 35 closer to less differentiated precursors than samples from day 25 and day 30 (Fig. 4a and Supplementary 1: HLCTC reduced matrix). Proteins marking a hepatocyte phenotype were consistently identified across replicates and displayed the greatest abundance on differentiation day 30 (Fig. 4b).

Differentiation of hiPSCs to HLCs requires the conversion from cell proliferation and migration to maturation. Quantification of selected proteins associated with cell growth was compared (Fig. 4c). Ki-67, a protein responsible for maintaining cell proliferation, was reduced in abundance from day 1 to 5 after which expression appeared stable. Proteins associated with cell cycle progression, which decreased throughout differentiation, included cyclin-dependent kinase-1, cyclin-dependent kinase 4, Geminin, and M-phase inducer phosphatase-3. The opposite was observed for cyclin-dependent kinase inhibitor 1B (CDKN1B, P46527) which governs G1 arrest by inhibiting cyclin E- and cyclin A-CDK2 complexes. These abundance changes demonstrated a reduction in proliferation during differentiation.

### Hierarchical clustering and markers throughout maturation

**Figure 5:**
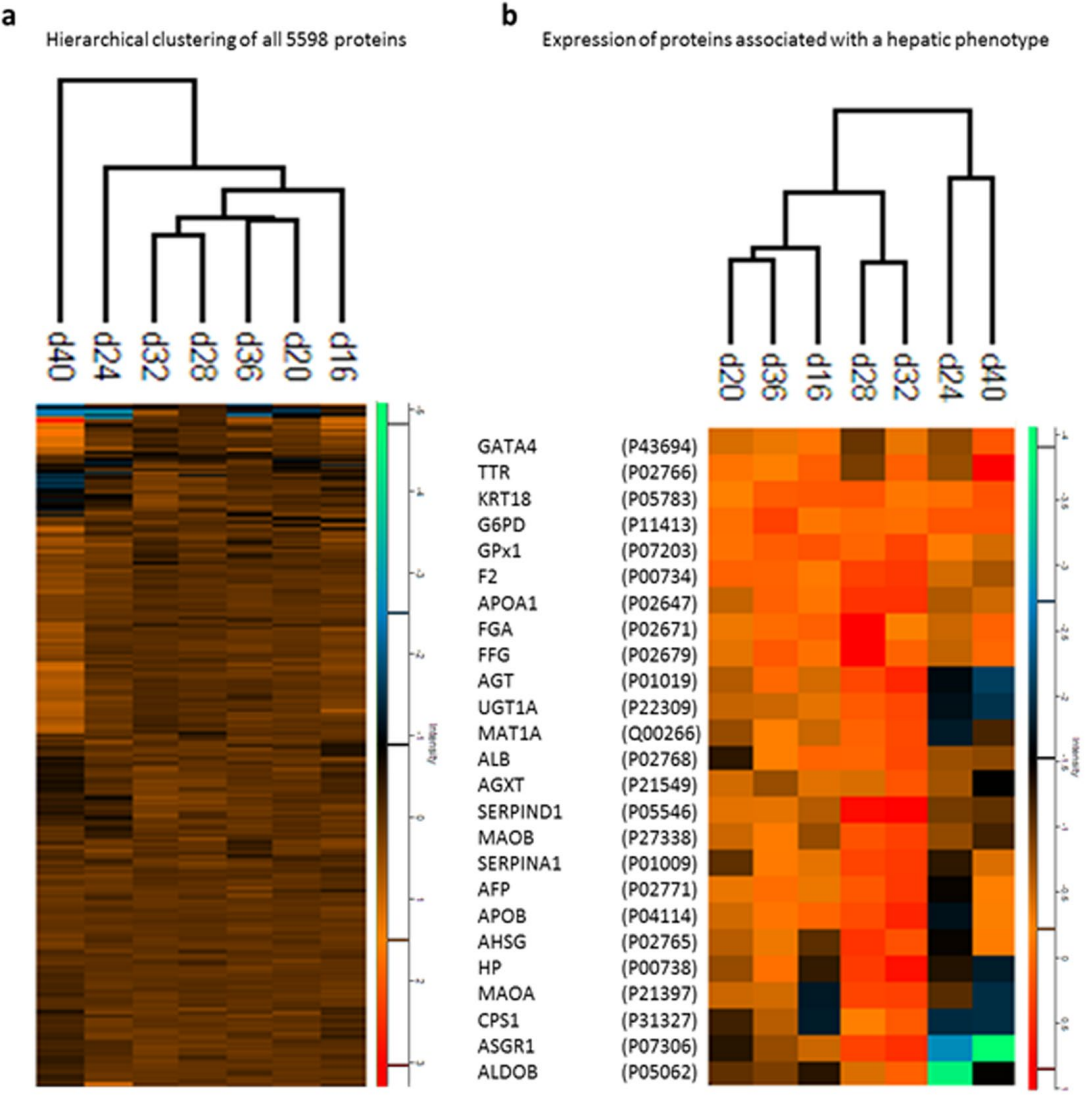
Hierarchical clustering of all proteins and hepatic marker subsets throughout HLC maturation. (**a**) Hierarchical clustering through HLC maturation and (**b**) Expression profiles of proteins marking a hepatic phenotype.

Hierarchical clustering for the maturation time course did not co-segregate chronologically (Fig. 5a and Supplementary 1: HLCLTC Hierarchical clustering) with day 24 and day 40 forming separate nodes. This could be a result of having only a single replicate for these two data points. However in PCA, HLCs on differentiation day 24 clustered well with samples from day 16 to day 36, which was not apparent for HLCs at day 40. During maturation, the characteristic hepatic markers investigated generally had greater abundance at day 28 and 32 (Fig. 5b). Here, as was evident in the complete differentiation time course, most phase I metabolizing enzymes were absent with none of the major CYP450 isoforms (CYP1A2, CYP3A4, CYP3A5, CYP2B6, CYP2C9, CYP2C19, and CYP2D6) identified confidently. Despite smaller fold-change differences in hepatic markers, some of which did not meet a distinct statistical cut-off, the two time course series were highly complementary. Reduced intensity differences could be as a result of the ratios being compared to already differentiated cells which have transitioned to early HLCs as opposed to the comparison relative to hiPSCs.

### Functional protein association networks

Hepatic lineages mature and acquire metabolic properties under a network of six functionally cross-regulating transcription factors with HNF4α controlling hepatic gene expression^30^. In addition, inductive cues promote sequential acquisition of nuclear transcription factor HNF4α in hepatic progenitors, AFP in hepatoblasts and albumin in hepatocytes^13^. This sequential acquisition, although not identical to *in vitro* differentiation, was used to select query proteins for assessing functional protein association networks.

The STRING database (http://string-db.org) was used to assess protein–protein interactions in functional protein association networks using core factors as query proteins. hiPSCs and anterior definitive endoderm query proteins were SOX2 and FOXA2 (Supplementary 2: Fig. S3). Despite a limited number of interactors being present, quantified protein interactors had higher abundance in hiPSCs. Proteins emerging during hepatic endoderm differentiation, APOA1 and APOB, had approximately half of the queried interactors quantified. A two-fold increase in expression of queried proteins APOA1, APOB, and interactors FGA and TTR were evident by day 10 which increased to a maximum around day 25 to 30.

Albumin, AFP and TTR were used as query proteins for hepatoblasts, which also had approximately half of the queried interactors quantified. While not demonstrating large fold-change differences across these proteins between day 25 and 35 of differentiation, the expression of hepatic proteins as well as key interactors demonstrates the progression through differentiation. HLCs had the most functional protein network interactors quantified (Supplementary 2: Fig. S4) while the relative abundance was maximally observed on day 28 or day 32. This query approach enabled extraction of quantitation information associated with key regulators which appear throughout differentiation.

## Discussion

Previously we have compared the proteome of the HLCs differentiated to day 35, under the same conditions characterized here, to other hepatic cell types^31^. While better correlated to PHHs than HepG2 monolayers or HepG2 3D spheroids, the phenotype of HLCs does not rival that of PHH donors. However, as no single hepatic model has yet been shown capable to mimic all *in vivo* hepatic functions, tracing the transitions of iPSCs into hepatic progenitors and further functionally qualifying HLCs provides an additional avenue for improving existing hepatic phenotypes. Functional characterisation and transcriptome analysis may not be sufficient for these qualifications making proteomic analysis such as these essential.

Although the correlation is not absolute, the coordinated pattern of embryonic liver development provides a guideline for *in vitro* hepatocyte differentiation. The liver develops following endodermal patterning via the ventral wall of the foregut endoderm, with differentiation of hepatoblasts into hepatic lineages and maturation completing developmental progression^30^. The ability of hierarchical clustering to sequentially segregate samples suggests a coordinated developmental pattern is successfully transitioning the proteome from undifferentiated precursors. The inability to segregate the maturation time course very specifically by collection time points could be as a result of reduced fold change differences in proteins once already expressing an HLC phenotype. In addition, the detection of lower abundance and hydrophobic proteins such as those required for metabolism, which are more likely to distinguish maturation, was likely limited by whole cell analysis.

Proteins used as markers for hepatocyte phenotypes were most abundant around day 30 of differentiation for the complete time course. If these proteins are accurate markers of hepatocytes, whether of an adult or foetal phenotype, then differentiating beyond 30 days may have a reduced hepatocyte-specific proteome explaining the clustering of day 35 with less differentiated counterparts. This phenomenon could not be inferred from a singular overview of the differentiation series which necessitated a similar comparison incorporating alternative time points. During maturation, hepatic markers were highly complementary to the differentiation time course and generally had the greatest abundance at day 28 and 32. Variance in the relative ratio of some proteins, despite undergoing adequate normalisation, points to concurrent and subsequent differentiations not always being tightly regulated. In spite of this, independently differentiated biological replicates of separate time courses suggests that maintaining the HLC proteome has definitive limits, which was previously demonstrated as a feature of this differentiation protocol^27^.

Correlation of the transcriptional fingerprint of differentiation from hiPSCs was not directly investigated here. However, there are 40 mRNAs suggested to be expressed solely in the liver when derived from hiPSCs^17^. Numerous proteins corresponding to this transcriptional fingerprint were identified. Serine-pyruvate aminotransferase, haptoglobin, s-adenosylmethionine synthase isoform type-1, prothrombin, angiotensinogen, heparin cofactor 2, and alpha-2-HS-glycoprotein were elevated at day 30 in the complete differentiation time course. Additionally, complement factor B, antithrombin-III, protein AMBP, complement 5, ribonuclease 4, beta-2-glycoprotein 1, fibrinogen alpha chain, vitronectin, fibrinogen gamma chain, and apolipoprotein A-II were also identified during maturation. Evidence of these correlations can be considered valuable support for this differentiation protocol.

hiPSCs differentiated using various strategies report numerous hepatocyte characteristics while the expression of mRNAs encoding phase I and phase II enzymes is largely incomplete^17^. Here there was a noteworthy absence of the major hepatic metabolizing enzymes, which is a potential limitation as six CYP450 enzyme isoforms collectively account for approximately 95% of CYP450-mediated reactions^32,33^. While limited in their expression of the panel of phase I and phase II metabolising enzymes raises some concerns over the phenotype of HLCs it cannot be established whether this was mostly due to limited protein abundance or as a result of the loss of hydrophobic peptides during proteomics sample preparation and analysis. Therefore, targeted proteomic investigation is required to further identify the extent of expression of the repertoire of metabolising enzymes.

An important characteristic essential to mimicking hepatocytes *in vivo* is the non-replicating state of PHHs, which is not achieved in immortalized cell lines. However, differentiation of hiPSCs to HLCs using this differentiation protocol demonstrated that cells undergo the conversion from proliferation and migration to maturation. Decreases in cell cycle regulating proteins, combined with the progressive increase in abundance of many hepatic proteins from hiPSC to HLCs, illustrates successful augmentation of the proteome.

## Conclusion

Mature HLCs are a valuable resource in regenerative medicine and pharmaceutical sciences. To the extent of our knowledge, there is no published data which has as comprehensively and systematically examined the proteome during differentiation of hiPSCs into HLCs. This proteomic data can be used as a resource for correlating *in vitro* differentiation with *in vivo* liver development and constructs a platform for optimisation of differentiation and the acquisition of mature HLCs. While limited in detection of low abundant proteins, protein localizations, and protein isoforms, this data provided the identification of thousands of proteins and created a proteomic reference platform through the transitions from pluripotency to HLCs. Despite the stochastic nature and variability of the proteome and differentiation, hepatic markers reproducibly demonstrated a trend towards maximum relative abundance between differentiation day 30 and 32. Characterisation of abundant proteins in whole cells provided evidence of time-dependent transition towards proteins corresponding with the functional repertoire of the liver and highlights the lengths to which HLCs have progressed from undifferentiated precursors.

## Materials and Methods

### Cell culture and differentiation

Human induced pluripotent stem cells (hiPSCs) were generated as previously reported^11,27,34^, where the chronological gene expression patterns through differentiation have been described. Prior to differentiation, hiPSCs were cultured in chemically-defined medium with polyvinyl alcohol (CDM-PVA: 250 ml Iscove’s Modified Dulbecco’s Media, 250 ml Ham’s F12 + GlutaMAX, 1% concentrated lipids, 0.7% insulin, 0.14% transferrin, 0.1% PVA, 1% penicillin/streptomycin supplemented with 10 ng/ml Activin A and 12 ng/ml FGF-2) and maintained in a tri-gas incubator (5% O_2_, 5% CO_2_, 90% N_2_) at 37 °C. Differentiation, using hiPSCs within 3–10 passages of thawing, was conducted as previously reported^27^ (Supplementary 2: Fig. S1).

### Sample collection

Cells were harvested for two proteomics series; a complete differentiation time course (hepatocyte-like cell time course: HLCTC) which included time points at days 1, 3, 5, 7, 10, 25, 30, and 35 from two separate differentiations with replicates at days 1, 5, 10, and 30. The second series constituted only days during HLC maturation (hepatocyte-like cell late time course: HLCLTC) which included time points at days 16, 20, 24 28, 32, 36, and 40 from two separate differentiations with replicates at days 16, 20, 28, 32, and 36. Cells were lysed using a Tris-based buffer (10 mM Tris-HCL (pH 8), 1 mM ethylenediaminetetraacetic acid, 0.5 mM ethyleneglycoltetraacetic acid, 1% Triton X-100, 0.1% sodium dodecyl sulphate, 0.1% sodium deoxycholate, 140 mM sodium chloride, and cOmplete™ protease inhibitor cocktail) assisted by ultrasonic disruption for 10 minutes at 4 °C.

### Protein digestion and TMT-labelling

Protein concentration was determined using the bicinchoninic acid assay with a 1:50 ratio of reagent B (4% copper II sulfate pentahydrate) to reagent A (2% sodium carbonate, 0.16% sodium tartrate, 0.9% sodium bicarbonate and 1% BCA; pH 11.25). Fifty micrograms of protein was reduced with 10 mM dithiothreitol at 37 °C and alkylated with 25 mM iodoacetamide for 2 hours at room temperature. Proteins were precipitated overnight at 4 °C with acetone and resuspended in 100 mM HEPES (pH 8.5). Samples were digested with 1.25 µg (1:40) sequence-grade modified trypsin for 1 hour at 37 °C. Additional trypsin (1:40) was added and digestion continued overnight at 37 °C. Tags were equilibrated to room temperature and resuspended in mass spectrometry-grade acetonitrile (41 µl per 0.8 mg tag). Digested peptides were clarified for 20 minutes at 16 000 *g* and labelled using TMT 6-plex reagent (Thermo Fisher Scientific) for 2 hours at room temperature under constant agitation (Labelling strategy: Supplementary 2: Table S1). Labelling was quenched with 8 µl of 5% hydroxylamine for 1 hour and further quenched overnight at 4 °C with dH_2_O. Labelled samples were combined and reduced to dryness by vacuum centrifugation.

### Solid phase extraction and peptide fractionation

Peptides were solubilized in dH_2_O with 0.1% TFA and loaded onto a conditioned SepPak C18 cartridge (100 mg). Desalting was conducted and peptides were eluted in 70% acetonitrile with 0.05% acetic acid. Eluents were dried and resuspended in 20 mM ammonium formate (pH 10) with 4% acetonitrile. Peptides were loaded via a single partial loop injection, onto a Waters ACQUITY UPLC BEH C18 column (130 Å, 2.1 mm × 150 mm, 1.7 µm), and fractionation conducted using a Waters ACQUITY system. Peptides were profiled at a flow rate of 0.25 ml/minute using an initial isocratic low organic phase (mobile phase A: 20 mM ammonium formate, pH 10; mobile phase B: 80% acetonitrile, 20 mM ammonium formate, pH 10). This was followed by a 50 minute linear gradient of increasing percentage mobile phase B (5-60%). Chromatography was monitored using a diode array detector scanning wavelengths between 200 and 400 nm. Fractions with peak peptide elution were dried and pooled into 15 samples using 0.1% formic acid.

### LC-MS/MS analysis

Samples were analysed using a Dionex Ultimate 3000 RSLCnano LC system and a Thermo Scientific Q Exactive Hybrid Quadrupole-Orbitrap Mass Spectrometer. Peptides (1–2 µg) were loaded onto an Acclaim PepMap 100 C18 pre-column (100 Å, 300 µm × 5 mm, 5 µm) using an Ultimate 3000 auto-sampler with 0.1% formic acid for 3 minutes at a flow rate of 10 µl/minute prior to elution onto a PepMap C18, EASY-Spray LC (100 Å, 75 µm × 500 mm, 2 µm) analytical column. Peptide separation was profiled at 300 nl/minute by applying a linear gradient of 4–40% using mobile phase A (H_2_O with 0.1% formic acid) and mobile phase B (80% acetonitrile, 20% H_2_O with 0.1% formic acid) over 100 minutes using a 120 minute run time. Mass spectrometry measured the mass-to-charge ratios (m/z) in positive ion data-dependent mode. Full MS scans were performed in the range of 380–1500 m/z at a resolution of 70,000 with an automatic gain control (AGC) of 5 × 10^6^ with a maximum injection time of 250 ms. Data dependent scans of the top 20 most abundant ions, with charge states between 2+ and 5+, were automatically isolated, selected and fragmented by higher energy collisional dissociation (HCD) in the quadrupole mass analyser. Dynamic exclusion was set at 60 seconds. HCD fragmentation was performed at a normalized collision energy (NCE) of 32.5% and a stepped NCE of 10% at a resolution of 17,500. The AGC, maximum injection time, first fixed mass and isolation window for MS2 scans was 5 × 10^4^, 150 ms, 100 m/z and 1.2 m/z respectively.

### Data processing

Raw data files were converted to MGF using ProteoWizard MSConvertGUI^35^ with filters for peak picking and a threshold count of 150. Peak lists were searched against a UniProtKB/Swiss-Prot human database (Homo sapiens, Canonical sequence, January 2016, Sequences: 20,194) using SearchGUI version 2.3.1^36^ with X!Tandem, MS-GF+ and Comet search engines. Post-processing of peptide-spectrum matches (PSM) for protein identification was done using Peptide Shaker version 1.7.3^37^. Search parameters included: minimum and maximum precursor mass of 300 and 900 Da respectively, precursor mass tolerance of 10 ppm, fragment mass tolerance of 0.2 Da and a maximum number of 2 missed cleavages. Fixed modifications included carbamidomethyl of cysteine, TMT 6-plex modification of lysine and peptide N-termini with variable modifications including oxidation of methionine and deamidation of asparagine or glutamine. Reporter version 0.2.13 (http://compomics.github.io/projects/reporter.html) was employed for deisotoping and relative quantification. PSM level ratios were estimated with the use of the median of all non-null intensities and a maximum likelihood estimator used for aggregation into peptide and subsequent protein grouping ratios.

### Data analysis and visualisation

**Figure 6:**
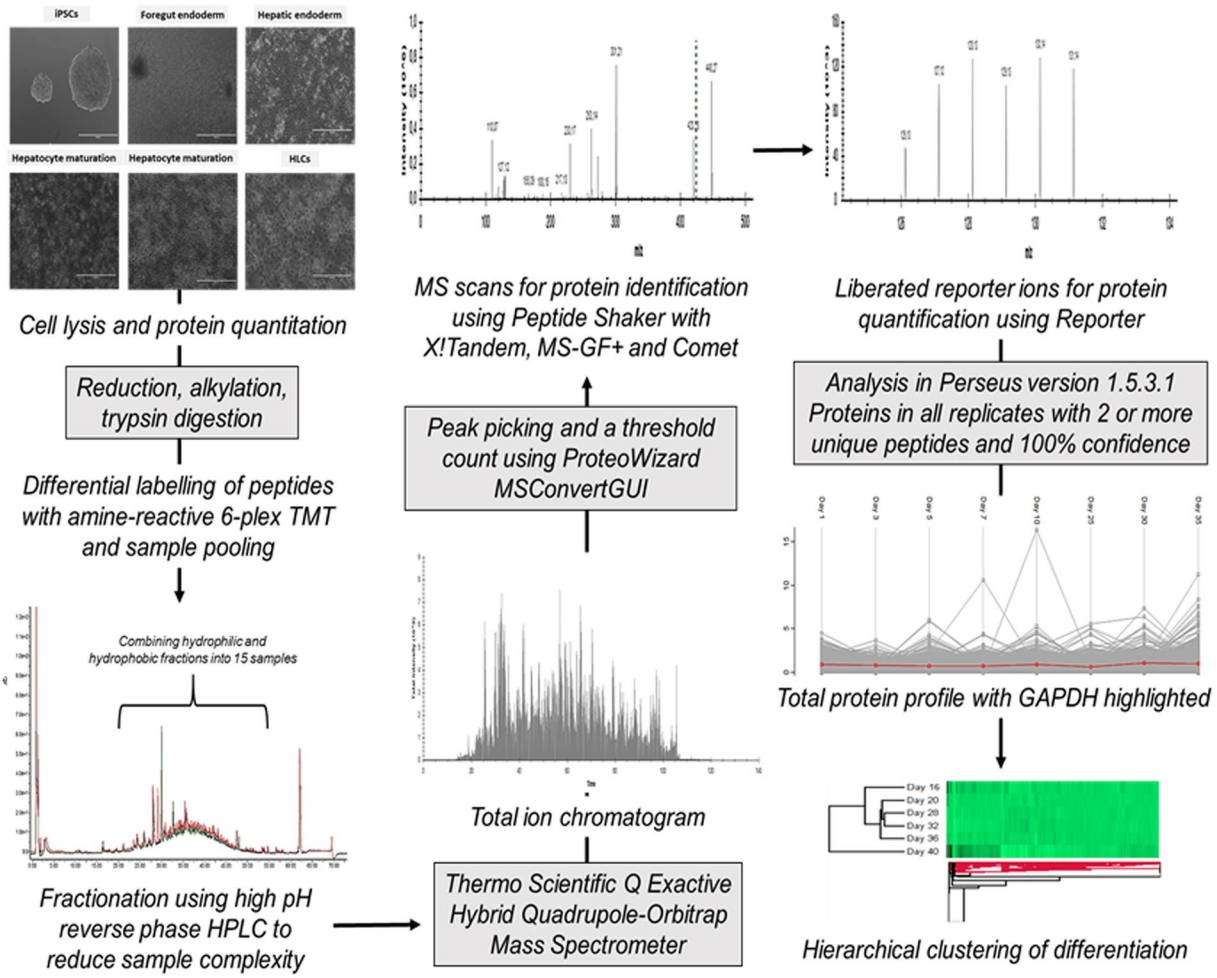
The experimental workflow from sample collection to analysis. Whole cell lysates were collected and 50 µg per sample was reduced, alkylated, and digested with trypsin. Peptides derived from each sample were differentially labelled with TMT 6-plex reagent and separated using reverse phase high performance liquid chromatography (HPLC). Fifteen pre-fractionated samples were analysed using liquid chromatography tandem-mass spectrometry (LC-MS/MS) with a linear gradient over 120 minutes. RAW files were converted to MGF with peak picking and threshold count filters. Peak lists were searched against a UniProtKB/Swiss-Prot human database using SearchGUI version 2.3.1 with X!Tandem, MS-GF+ and Comet search engines. Identification and quantification were done using Peptide Shaker version 1.7.3 and Reporter version 0.2.13 respectively. Data analysis and visualisation was conducted using Perseus software.

Proteins present in respective replicates and identified with 100% confidence using a minimum of 2 unique peptides were analysed in Perseus version 1.5.3.1 (Max Planck Institute of Biochemistry). Protein profiles were generated and clustering established based on Euclidean distances from reference profiles. Protein ratios, using the average across replicates, were expressed relative to controls. K-mean pre-processing and average linkages were used for hierarchical clustering. Where applicable, multi-sample testing was conducted using ANOVA with a permutation-based false discovery rate (FDR) for truncation, and a FDR of 0.01 with 250 randomizations. PCA was done using a Benjamini-Hochberg cut-off method with a FDR of 0.01. Proteins were annotated for gene ontology (GO) biological processes (BP), molecular functions (MF), cellular components (CC) as well as Reactome and KEGG pathway identifiers. Data was interrogated for protein-protein interactions using STRING (Search Tool for the Retrieval of Interacting Genes/Proteins). Proteins associated with each phase of differentiation were selected and 10 interactors with a 0.900 minimum confidence interaction score were assessed for their identification in the data set. The entire experimental workflow is detailed in Fig. 6.

## Supplementary information

Dataset 1

Dataset 2

## Data Availability

Further details pertaining to protocols, materials and data are available from the corresponding author.
