# Supplementary material for: A proteomic time course through the differentiation of human induced pluripotent stem cells into hepatocyte-like cells
Source: Sci Rep. 2019 Mar 1;9:3270. doi: 10.1038/s41598-019-39400-1 (PMC6397265; doi:10.1038/s41598-019-39400-1)
Supplement: Supplementary file 2 — Dataset 2 [file 41598_2019_39400_MOESM2_ESM.pdf]

Manuscript title:

A proteomic time course through the differentiation of human induced pluripotent stem cells into hepatocyte-like cells

Manuscript ID: SREP-18-37419

Authors and affiliations:

Tracey Hurrell[1,4] (tracey.hurrell@ki.se)*

Charis-Patricia Segeritz[2] (charis.walko@gmail.com)

Ludovic Vallier[2,3] (lv225@cam.ac.uk)

Kathryn S. Lilley[4] (k.s.lilley@bioc.cam.ac.uk)

Allan D. Cromarty[1] (duncan.cromarty@up.ac.za)

[1]Department of Pharmacology, Faculty of Health Sciences, School of Medicine, University of Pretoria, Private Bag X323, Arcadia, 0007, South Africa

[2]Wellcome Trust–Medical Research Council Cambridge Stem Cell Institute at Anne McLaren Laboratory
Laboratory for Regenerative Medicine, Department of Surgery, University of Cambridge, Robinson Way, Cambridge, CB2 0SZ, United Kingdom

[3]Wellcome Trust Sanger Institute, Hinxton, United Kingdom

[4]Cambridge Centre for Proteomics, Department of Biochemistry, University of Cambridge, Tennis Court Road, Cambridge CB2 1QR, United Kingdom

[d]Cambridge Centre for Proteomics, Department of Biochemistry, University of Cambridge, Tennis Court Road, Cambridge CB2 1QR, United Kingdom

Correspondence:

Tracey Hurrell
Section of Pharmacogenetics
Department of Physiology and Pharmacology
Karolinska Institutet
Solnavägen 9, Biomedicum
171 77 Stockholm, Sweden
tracey.hurrell@ki.se
+46-(0)8-524 877 62

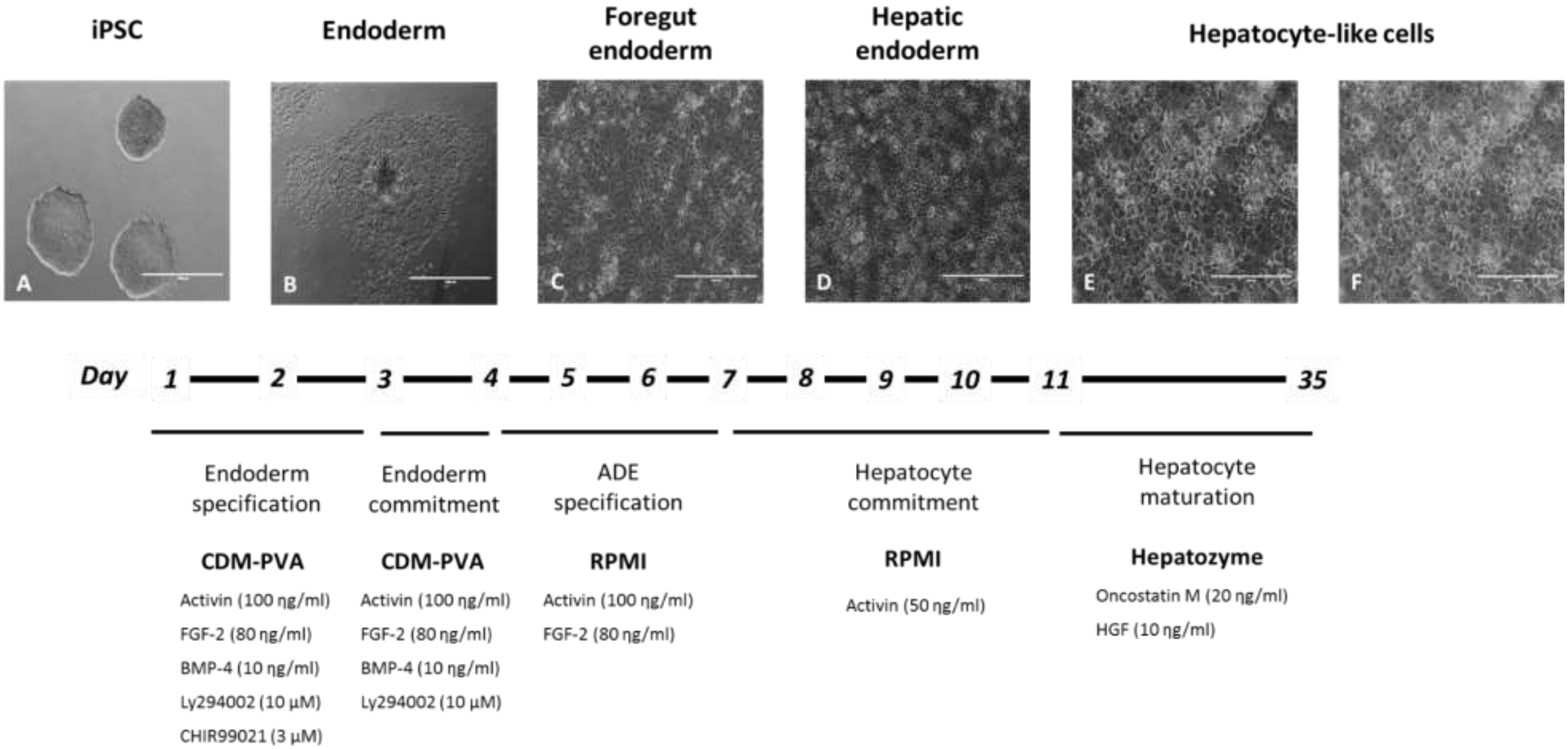

Supplementary Figure S1: ***Phase contrast images (EVOS FL Cell Imaging System) showing differentiation of human induced pluripotent stem cells (iPSCs) into hepatocyte-like cells.*** *A) Human iPSCs organised in tightly packed colonies, B) Definitive endoderm specification with cellular migration from the colony, C) Anterior definitive endoderm (ADE) cells, D) Hepatic endoderm, and E-F) Hepatocyte-like cell maturation. CDM-PVA: chemically defined medium with polyvinyl alcohol, FGF: fibroblast growth factor, BMP: bone morphogenic protein, HGF: hepatocyte growth factor. Scale bar: 400 μm. Redrawn from (Hannan, Segeritz et al. 2013).*

Supplementary Table S1: Tandem mass tags used for proteomic time courses

	Differentiation time course from day 1 to day 35 (HLCTC)		Hepatocyte maturation time course from day 16 to day 40 (HLCLTC)	
**TMT label**	**Replicate 1**	**Replicate 2**	**Replicate 1**	**Replicate 2**
**126**	iPSC (Day 1)	iPSC (Day 1)	HLC (Day 36)	Late-HLC (Day 40)
**127**	Endoderm (Day 5)	Endoderm (Day 3)	Maturation (Day 32)	HLC (Day 36)
**128**	Progenitors (Day 10)	Endoderm (Day 5)	Maturation (Day 28)	Maturation (Day 32)
**129**	Maturation (Day 25)	Progenitors (Day 7)	Maturation (Day 24)	Maturation (Day 28)
**130**	Maturation (Day 30)	Progenitors (Day 10)	Maturation (Day 20)	Maturation (Day 20)
**131**	HLC (Day 35)	Maturation (Day 30)	Maturation (Day 16)	Maturation (Day 16)

Supplementary Table S2: List of markers throughout hepatocyte differentiation phases by Schwartz *et al.* and the correlation with the proteomic time course replicates

**Differentiation phase**	**Molecular marker**<br>Proteins in bold were identified in both replicates of at least one time course<br>Proteins underlined were identified in at least one replicate of either time course
Pluripotency	NANOG, OCT4, SOX2, SSEA3/4, TRA 1-60
Anterior definitive endoderm	*Cerberus*, CXCR4, *FGF17*, *FOXA2*, Goosecoid, HHEX, MixL1, ***SOX17***, GATA4
Hepatic endoderm	***APOA1, APOB***, BMP6, *Cerberus*, CXCR4, DUSP6, *FOXA2*, ***GATA4***, HHEX, ***HNF4α***, **SOX-17**, *TBX3*
Hepatoblasts	***APOA1, APOB, AFP, ALB***, Decorin, FOXA2, GATA4, GSTA1, HNF1α, ***HNF4α***, HHEX, KRT19, **SOX17**, *TBX3*, ***TTR***
Adult hepatocytes	***A1AT, ALB, AFP, APOA1, APOB***, APOF, ***ASGR1***, BSEP, ***CK8, CK18***, CPS1, *CYP1A2*, *CYP2A6*, *CYP2B6*, *CYP2C9*, *CYP2C19*, *CYP2D6*, *CYP3A4*, CYP3A7 (low), Decorin, FOXA2, ***GATA4***, *GSTA1*, HNF1α, ***HNF4α***, HHEX, ***MAOA***, ***MAOB***, ***MRP2,*** TBX3, ***TTR, UGT1A1***
Fetal hepatocytes	***A1AT, APOA1, APOB, AFP, CK8, CK18, CYP3A7,*** Decorin, FOXA2, ***GATA4***, *GSTA1*, HNF1α, ***HNF4α***, HHEX, ***KRT19,*** TBX3, **TTR**

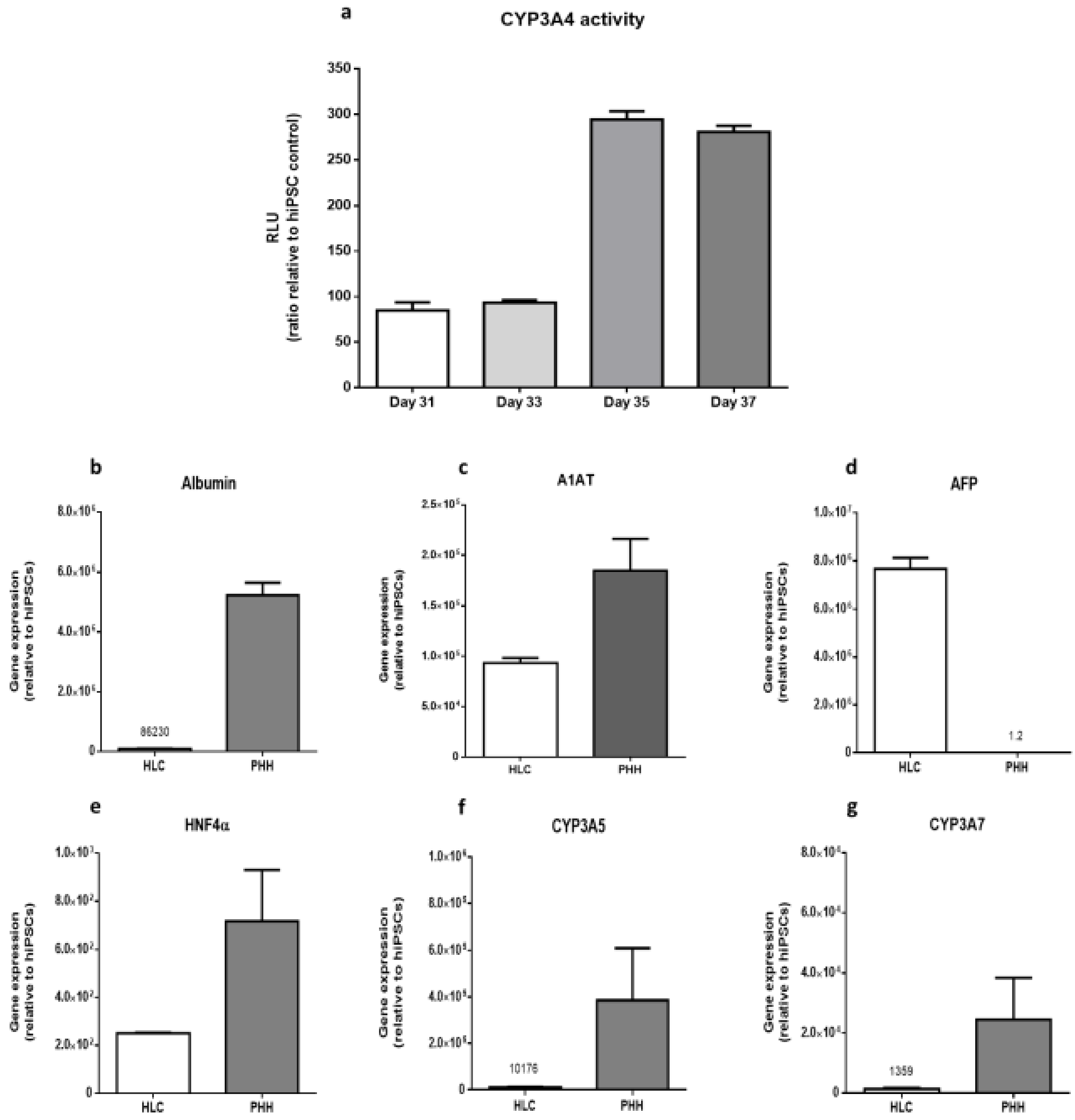

Supplementary Figure S2: ***Assessment of the functionality and expression of common hepatic markers.*** *a) CYP3A4 activity (P450-Glo CYP3A4 assay) increased overtime in HLCs (n=3) from day 31 to day 37 compared to hiPSCs. qPCR data comparing expression in iPSCs to differentiated HLCs (n=3) and isolated PHHs (n=3) for b) Albumin, c) α1-antitrypsin (A1AT), d) α-fetoprotein (AFP), e) hepatocyte nuclear factor 4α (HNF4α), f) CYP3A5, and g) CYP3A7. Basal transcript level of AFP was low in PHH but highly expressed in HLCs. Despite low gene expression of HNF4α, this coupled to detection of albumin and AFP suggests transition to hepatic progenitors.*

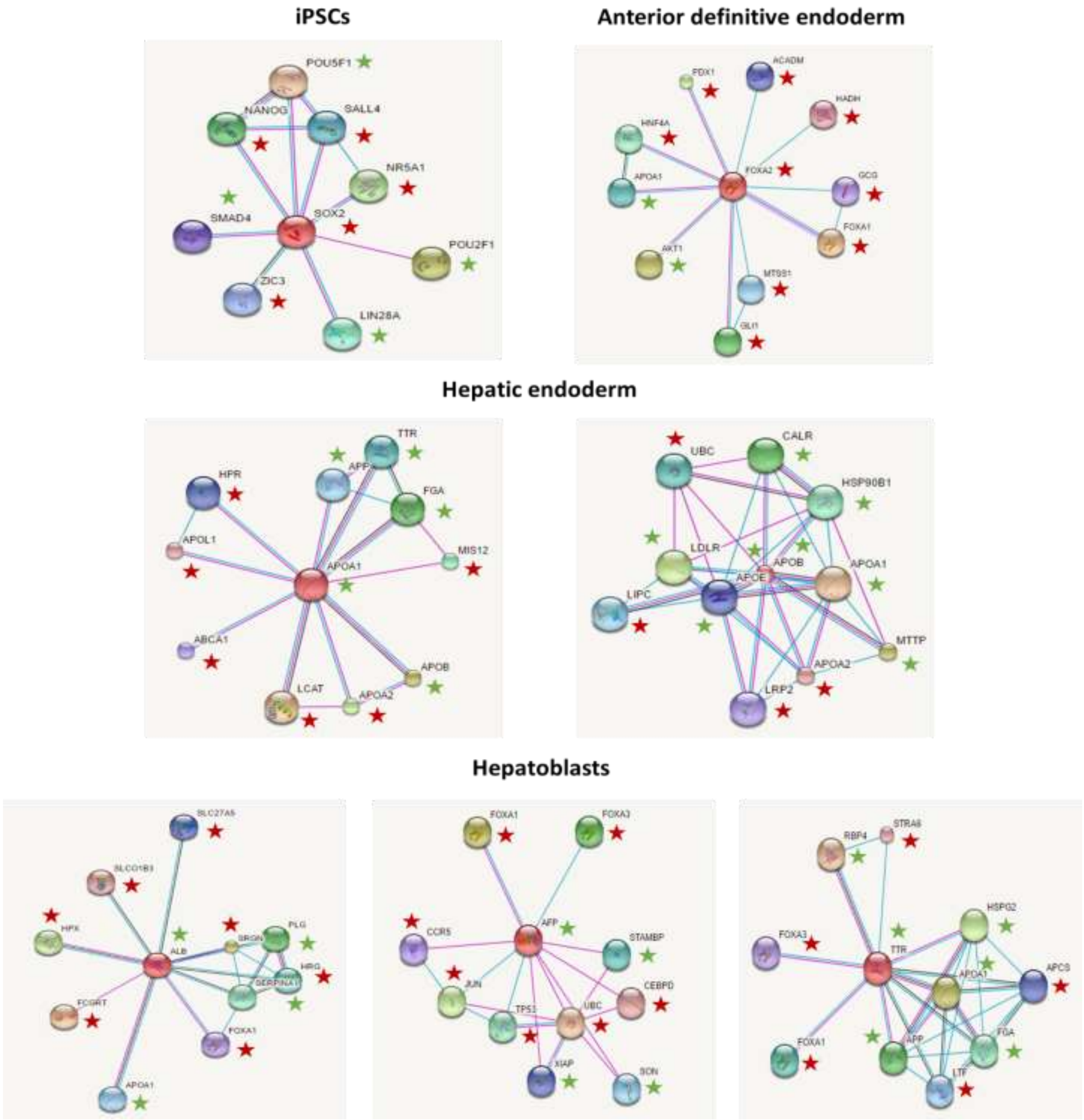

Supplementary Figure S3: ***Functional protein association networks in hiPSCs, anterior definitive endoderm, hepatic endoderm and hepatoblasts.*** *Query proteins (red circle) are linked to known interactors from curated databases (blue line) or have been experimentally determined (pink line). Interactors which were present in the HLCTC data set are indicated with a green star while those absent are indicated with a red star. Query proteins included SOX2 for iPSCs, FOXA2 for anterior definitive endoderm, APOA or APOB for hepatic endoderm and ALB, AFP or TTR for hepatoblasts.*

Hepatocyte-like cells

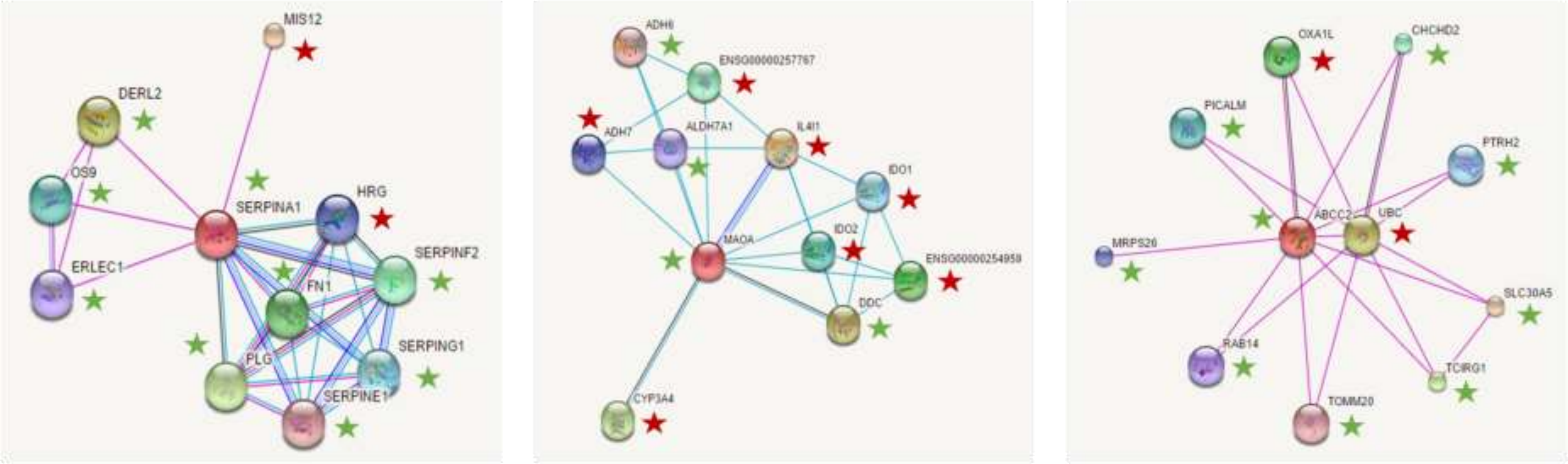

Supplementary Figure S4: ***Functional protein association networks in hepatocyte-like cells.*** *Query proteins (red circle) are linked to known interactors from curated databases (blue line) or have been experimentally determined (pink line). Interactors which were present in the HLCLTC data set are indicated with a green star while those absent are indicated with a red star. Query proteins included SERPINA1, MAOA orABCC2.*
